# Childhood ADHD and treatment outcome: the role of maternal functioning

**DOI:** 10.1186/s13034-018-0234-3

**Published:** 2018-06-14

**Authors:** Pernille Darling Rasmussen, Ole Jakob Storebø, Yael Shmueli-Goetz, Anders Bo Bojesen, Erik Simonsen, Niels Bilenberg

**Affiliations:** 1 Child and Adolescent Psychiatric Department, Region Zealand, Denmark; 2 Psychiatric Research Unit, Region Zealand, Denmark; 3 Child and Adolescent Psychiatric Department and, Psychiatric Research Unit, University of Southern Denmark, Odense, Denmark; 4 Institute of Psychology, University of Southern Denmark, Odense, Denmark; 5 Department of Clinical Medicine, University of Copenhagen, Copenhagen, Denmark; 6 Anna Freud National Centre for Children and Families, London, UK; 7 Ny Østergade 12, 4000 Roskilde, Denmark

**Keywords:** ADHD, Attachment, Resilience, Maternal functioning, Developmental outcome

## Abstract

**Background:**

Relatively little is known about the role of maternal functioning in terms of attention deficit hyperactivity disorder (ADHD) symptoms, attachment style and resilience as predictive factors for treatment outcome when offspring are diagnosed with ADHD.

**Objective:**

To investigate whether maternal functioning is associated with treatment outcome in children with ADHD.

**Methods:**

The study formed part of a larger naturalistic observational study of children with ADHD. A battery of self-report measures was used to assess selected factors in maternal functioning at the point of referral (baseline data); adult ADHD-symptoms, adult attachment style and adult resilience. Associations between these domains and child treatment response were subsequently examined in a 1-year follow up.

**Results:**

Maternal ADHD-symptoms and degree of resilience were significantly correlated to symptom reduction in offspring diagnosed with ADHD. However, the association between maternal attachment style and child treatment response as measured by the ADHD-RS did not reach statistical significance.

**Conclusion:**

To our knowledge, this is the first study to consider potential protective factors along with risk factors in maternal functioning and the impact on child treatment outcome. The study contributes to our knowledge of the potential role of maternal functioning in treatment outcome for children with ADHD.

**Electronic supplementary material:**

The online version of this article (10.1186/s13034-018-0234-3) contains supplementary material, which is available to authorized users.

## Background

Attention deficit hyperactivity disorder (ADHD) is the most commonly occurring neurodevelopmental disorder in childhood with a prevalence ranging from 3 to 5% and symptoms often continuing into adulthood [1]. It is characterized by a number of core symptoms including inattention, hyperactivity and impulsivity [2]. Both the DSM-5 and ICD-10 criteria require excessive inattention, hyperactivity, and impulsivity to be inconsistent with the developmental level and to be pervasive [3, 4]. According to the DSM-5 three presentations of ADHD, differentiated on the basis of symptom load, are commonly referred to: combined-type, inattentive-type and hyperactive/impulsive-type. For a formal diagnosis, the symptoms have to be present for at least 6 months and result in impairment in more than one setting before the age of 6 (ICD-10) or 12 (DSM-5) (WHO 1992, [4]).

### Maternal ADHD, attachment style, and resilience

The etiology of ADHD is multifactorial, as both genetic and environmental factors have been evidenced in the development of ADHD [5, 6]. For example, a relative with ADHD [7], an increase in Copy Number Variation (CNV, [8]), prematurity [9] and some form of neglect (Thapar et al. 2012) have all been implicated. Moreover, numerous studies have found ADHD to be associated with a poor prognosis (e.g. more divorces, higher rates of substance abuse disorders in adulthood, and increased mortality rate) [11–13]. Furthermore, the prognosis of ADHD worsens in the presence of comorbidity [11, 14]. Parent and child ADHD are found to be significantly associated, as well as parent and child conduct problems [15]. However, factors associated with the developmental progression and the long-term prognosis of ADHD are not fully understood [15–17] and hence, a greater focus on the developmental progression of ADHD is required. Whereas studies converge in proposing that parental psychopathology poses a high risk of transmission to offspring, relatively little is known about the role of maternal functioning in terms of attention deficit hyperactivity disorder (ADHD) symptoms, attachment style and resilience as predictive factors for treatment outcome when offspring are diagnosed with ADHD.

Maternal ADHD may be a potential risk factor in the development of offspring ADHD [18–21]. Moreover, in a recent study on associations between parental psychiatric disorders and offspring ADHD, maternal diagnosis showed stronger associations with child ADHD than paternal diagnosis [22]. Despite research establishing a link specifically between parental ADHD and parenting, measured as the level of home chaos, and parenting practices assessed through self-reports [23], to date there have been no studies investigating the role of maternal ADHD symptomatology as a prognostic factor in offspring treatment outcome.

In addition to the contribution of maternal ADHD symptoms to the increased risk of offspring developing ADHD, the quality of the mother–child relationship has also come under scrutiny. Indeed, both ADHD and attachment have been proposed as risk factors [24]. Reviewing the literature on ADHD and attachment, we found a clear association between ADHD and insecure attachment. When one condition was present, this increased the risk of developing the other. This underlines that ADHD and insecure attachment may constitute mutual risk factors [25]. In a recent comprehensive review of parental self-reported attachment style and caregiving, adult attachment security was consistently associated with more positive parenting whereas insecurity was related to more negative parenting [26]. These findings underscore the importance of investigating maternal ADHD-symptoms and attachment style as part of a broad assessment of maternal functioning. Alongside the contribution of adult attachment to maternal functioning, a growing body of research suggests that resilience is also a key factor, with greater resilience associated with psychological adaptation and functioning in the face of adversity [27–29]. Broadly, resilience theory focuses on understanding healthy development in the face of risk, and on strengths as opposed to weaknesses. It has been defined as a “pattern of positive adaptation in the context of past or present adversity” [30]. Critically, resilience does not suggest the absence of adversity or risk, but rather highlights the presence of protective processes leading to healthy adaptation. Whilst definitions and measurement of resilience vary considerably from study to study and the scientific value of the concept generally has been debated and challenged [31] resilience has been found to influence treatment response across different manifestations of adversity. These include chronic illness, psychiatric disorders and school bullying [32, 33].

In this study, the focus is on the significance of maternal resilience for parenting in situations where the child needs extra support.

### The current study

The aim of the present study was to examine associations between mothers’ functioning and treatment response in their children diagnosed with ADHD who are receiving care as usual.

We hypothesized that maternal self-reported ADHD symptoms; self-reported attachment style and maternal resilience would all be significantly correlated with treatment outcome. More specifically, we anticipated the following; (1) higher maternal ADHD-symptom scores would be associated with lower ADHD symptom reduction in offspring within the first year of treatment. (2) Higher scores for self-reported anxiety or ambivalence on the attachment style questionnaire are associated with lesser symptom reduction in offspring. (3) A higher degree of self-reported maternal resilience is associated with better treatment response in offspring diagnosed with ADHD.

## Methods

The current study was part of a naturalistic observational study exploring different aspects of maternal functioning expected to influence treatment response in children diagnosed with ADHD.

### Participants

The families participating were recruited from two child psychiatric outpatient clinics in Region Zealand, Denmark. The four interviewers participating were the same at the two sites; two conducting maternal attachment interviews and two conducting child attachment interviews. Sixty-seven (N = 67) child-mother dyads were included in the follow up.

Of the 67 mothers, 64 (95.5%) provided adequate responses on the baseline questionnaires to include for analysis. Three dyads were excluded from the analysis as they had not responded to all the questions. Age, gender distribution and the diagnoses of the children, along with other sample characteristics of parents and children can be seen in Table 1a and b. The mean age was 9.1 years, with children ranging in age from 7 to 12 years. A large proportion of the children came from one-parent households (53%) with the rest living together with both biological parents. Fifty-four (84.4%) responders reported a history of psychiatric illness in parents, siblings or grandparents. The children received *Care as usual* according to national guidelines [34]. At 3 months follow up, 43.8% of the children received medical treatment increasing to 70.3% at 6 months follow up, and 71.9% at 9 and 73.4% 12 months follow up. There were no reports of children dropping out of treatment during the 1-year follow up.

**Table 1:** (a) Child characteristics, (b) family characteristics

	n	%
a		
Gender		
Male	46	71.88
Female	18	28.13
Age at baseline—mean (SD) = 9.1 (1.3)		
ADHD subtype		
Combined type	50	78.1
Inattentive type	14	21.9
Comorbidity		
None	51	79.7
Oppositional defiance disorder	5	7.8
Autism spectrum	4	6.3
Other	4	6.3
b		
Parent job status		
Unemployed	15	23.4
Employed or student	42	65.6
No information	7	10.9
Household type		
Nuclear family	30	46.9
Split family	34	53.1
Psychiatric history		
No	9	14.1
Yes	54	84.4
No information	1	1.6

## Measures

### Maternal measures

#### Resilience in Adults Scale (RSA; [35, 36])

Maternal resilience was measured by the Resilience in Adults Scale (RSA)—a 33-item self-report scale for measuring resilience in adults. The scale covers six dimensions assessing protective factors at the personal level as well as at a family and a social level. The RSA is based on a seven point semantic differential scale with a positive attribute at the high end of the scale and a negative attribute at the low end of the scale. An example of a positive attribute in Personal Competence is ‘‘I know if I continue, I will succeed’’. Half of the items are reversed to reduce acquiescence biases. The maximum score achievable is 231 (high resiliency) and the lowest possible score is 33. The scale has been found reliable for distinguishing clinical samples versus the normal population [37–39].

#### The Adult ADHD Self-Report Scale (ASRS 1.1; [40])

Maternal ADHD symptoms were measured using the Adult ADHD Self-Report Scale (ASRS 1.1, short version of 6 items, two dimensions). The ASRS 1.1 is a 6-item screening version of a longer 18-item scale. It is used to assess ADHD symptoms in the previous 6 months and includes four items on inattention and two on hyperactivity. Symptoms are rated on a 5-point response scale (never—scored 0, rarely—1, sometimes—2, often—3, and very often—4). The total score ranges from 0 to 24. In a convenience subsample of subscribers to a large health plan in the US, the ASRS Screener was administered twice to assess test–retest reliability and then a third time together with a clinical interviewer. The ASRS Screener was found to be in high concordance with clinician diagnoses. Internal consistency reliability was in the 0.63–0.72 range and test–retest reliability (Pearson correlations) in the 0.58–0.77 range [41].

#### Experiences in Close Relationships Scale—Revised (ECR-R; [42])

Maternal attachment style was measured using the Experiences in Close Relationships Scale—Revised (ECR-R)—36 items in total, 18 items assessing romantic attachment anxiety (model of self) and 18 items assessing romantic attachment avoidance (model of others). The questionnaire is a widely used self-report measure of adult romantic attachment and is based on the theoretical assumption that anxiety and avoidance are the two fundamental dimensions underlying attachment. Items are rated on a 7-point Likert scale ranging from 0 (*strongly disagree*) to 6 (*strongly agree*). In a study by Sibley and colleagues psychometric properties (i.e., the test–retest reliability, convergent, and discriminant validity) of the ECR-R were investigated and documented [43]. For the current study, participating parents were instructed to include previous and current relationship experiences when answering the questions.

### Child measures

#### K-SADS-PL; [44, 45]

The children were screened using the Schedule for Affective Disorders and Schizophrenia for School-aged Children, Present and Lifetime Version (K-Sad-PL).

The interview is a valid and widely established diagnostic measure and it allows clinicians to classify children and adolescents with respect to their psychiatric diagnoses according to DSM-IV systems. In a recent study, the convergent and divergent validity of two diagnostic groups, anxiety disorders and ADHD was investigated. It was concluded that the K-SADS-PL generates valid diagnoses of anxiety and ADHD including the predominately inattentive subtype [46].

#### ADHD-rating scale (ADHD-RS; [47])

Child ADHD symptoms were measured using the revised ADHD-RS [48, 49] (translated into Danish and validated) to assess the severity of ADHD symptoms in children aged 4–17 years. It consists of 26 items loading on to attention deficit, hyperactivity/impulsivity and behavioral problems. Each item is rated from 0, denoting never/rarely, to 3, denoting very often. The total score ranges from 0 to 78. The schedule can be administered by teachers and parents and has been found to be valid and reliable when measuring symptom load in a clinical population [47, 50].

### Procedure

Initial identification was based on referral diagnosis based on ICD-10 (ADHD: combined-type, inattentive-type and hyperactive/impulsive-type) followed by a clinical evaluation in order to establish whether the family met the inclusion criteria.

In order to lower the risk of selection bias, we applied consecutive recruitment, as all the families with children in the appropriate age range presenting the relevant referral diagnosis, were invited to participate.

**Fig. 1:**
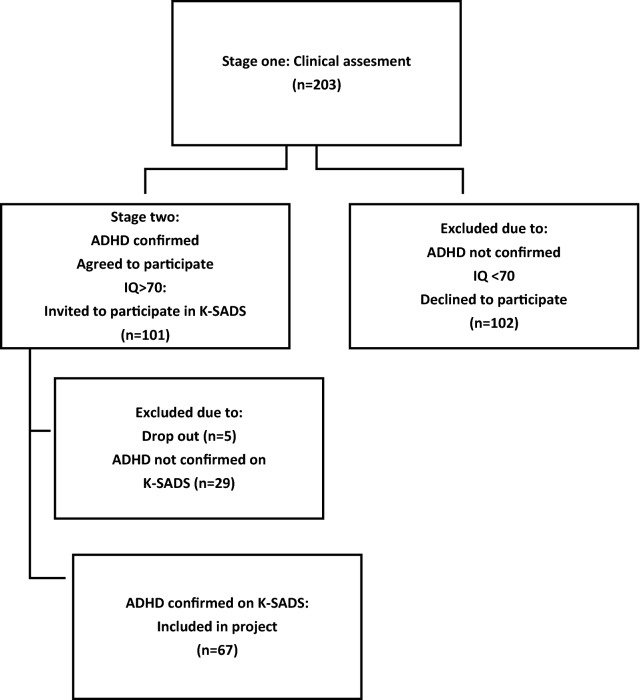
Two-stage inclusion process

The data obtained through questionnaires and interviews in the two-stage inclusion process and a 1-year follow up period were analysed using structural equation modeling and mixed effects modeling for repeated measures (Fig. 1).

During the recruitment phase, 203 children were referred with a possible diagnosis of ADHD. Only children in the age range of 7–12 representing middle childhood were subsequently invited to participate in the study. Exclusion criteria further included children who were adopted or living in foster care and children with major handicaps, such as hearing impairment or learning disabilities, preventing them from participating in and completing the interviews. Children who during the inclusion procedure were suspected of psychosis or who had an IQ below 70 were excluded.

Those who met the above criteria were invited to participate and were given further information about the study in a telephone call by the 1st author. The second stage of inclusion involved a formal assessment of ADHD diagnosis, using the Schedule for Affective Disorders and Schizophrenia for School-aged Children, Present and Lifetime Version (K-Sad-PL) [44, 45].

**Fig. 2:**
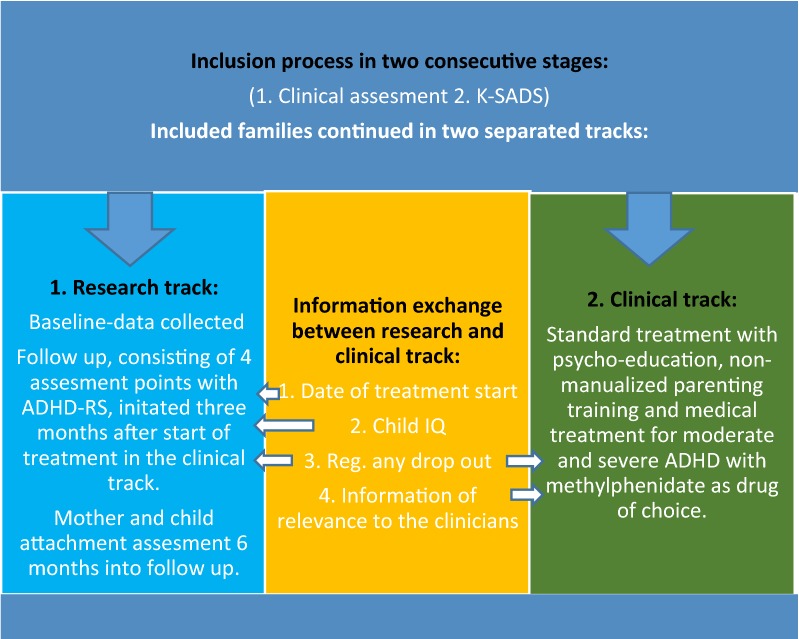
Overview of clinical and research track

Only patients assigned an ADHD/ADD diagnosis according to DSM-V with ADHD (314.01) or ADD (314.00) in step two were included for follow up. The included families attended both a clinical and a research track separated from each other (Fig. 2).

Apart from exchanging information on treatment initiation, child IQ and in the event of drop out from medical treatment, the research project was separated from the clinical track.

#### Care as usual

The children included all received *care as usual* consisting of standard medical treatment with methylphenidate as first choice for moderate and severe ADHD. The interventions were standardized and formalized according to national guidelines [34].

In Denmark, clinical guidelines adhere to recommendations from NICE-guidelines [51]. Recommendations are divided into three age groups; pre-school children, school-age children and young people with ADHD and moderate impairment and school-age children and young people with severe ADHD and severe impairment. For the school-aged children/moderate impairment, group-based parent-training/education programs are recommended as first-line treatment. Drug therapy is recommended only for those with “severe symptoms and impairment or for those with moderate levels of impairment who have refused non-drug interventions, or whose symptoms have not responded sufficiently to parent-training/education programs”. When the child has severe ADHD/impairment, medical treatment is recommended as the first line. All the participating children in this study were school-aged children. The research project was not involved in choosing treatment, but was informed from the clinical track that no families received additional treatment initiatives than that described in the standard program. The compliance rate was excellent, as there was no reports of dropout from treatment during the 12 months of follow up.

#### Baseline screening of parents

Mothers were asked to complete questionnaires regarding themselves. The questionnaires screened for ADHD symptoms using the ASRS 1.1, assessed attachment styles as relating to current romantic relationships using the ECR, and measured the degree of resilience using the RSA. All questionnaires were sent as links by e-mail from the online data managing system Easytrial.

#### Easytrial ©

In order to obtain a high response rate in the follow-up period, we used the Easytrial© online clinical data management system. All questionnaires from the baseline procedure as well as the follow up sequence were sent out as links to the parents by e-mail. The system provided an opportunity to monitor non-responders and send out ‘gentle reminders’. Responses were collected directly in a secured database compliant with good clinical practice (GCP) and database security legislation.

#### Follow up

**Fig. 3:**
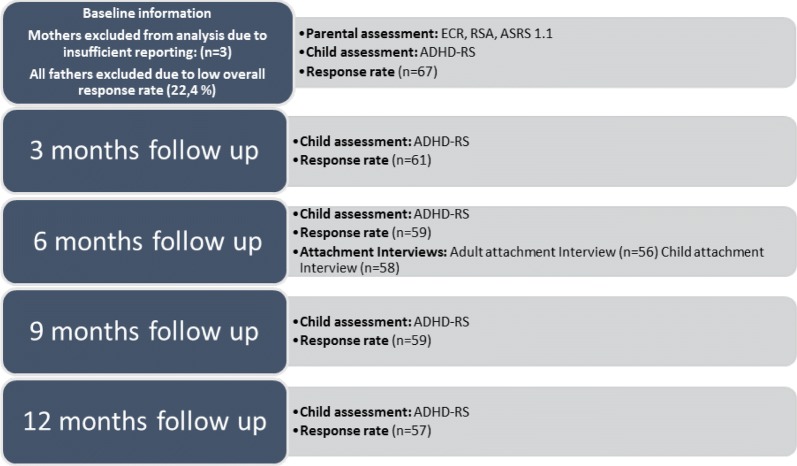
Details of research track during follow up

The families progressed to the follow up 3 months after the initial assessment (Fig. 3).

This delay was to ensure that the assigned treatment had been initiated. The attachment assessment was conducted at the child psychiatric clinics by four trained interviewers. The interviewers were not involved in treatment and had no prior knowledge of the families. The two interviewers of the mothers were blinded to results of the child assessment and vice versa.

The follow up consisted of four ADHD-symptom screenings completed every third month within the first year of inclusion. The ADHD-RS was sent to the parents for assessment of ADHD-symptom load in their children. The observed change (slope) in ADHD-RS from baseline to the 12 month follow up represents the primary outcome’.

In one-parent households, the schedule was sent to the household in which the child was residing. More than half (53%) of the families were one-parent households and only in one family was the child residing with the father.

### Data analysis

Treatment response was measured as the change in the ADHD-RS scale over the course of the 12 months of follow up. The change was calculated as the best fitting linear slope for each patient with time as a predictor of ADHD-RS. Due to some missed self-reports, this slope was in some cases based on only two observations in the follow up sequence with the other three missing (baseline and four follow up ADHD-RS measurements). A random effects linear regression was used to predict the slope of ADHD-RS change for each child. This slope is used as a continuous measure of treatment response. For instances of missing baseline measurements, the baseline score was approximated by an intercept estimate based on other measurements available. Data on missed self-reports is provided in Table 2. Random intercepts and slopes were predicted using a random effects linear regression model, with repeated measures (baseline and four follow up ADHD-RS measurements) nested in patients. In a set of linear regression models, each patient’s predicted slope (change in ADHD-RS) was regressed on ECR, RSA and ASRS with and without adjustment for gender, age, family type, medical treatment status, familial psychiatric history and the number of missing data points out the five follow up measurements. This last element is included to estimate and control for any possibly association between non-attendance and treatment effect. Control variables and background variables are summarized using frequency tables, means and standard deviations.

**Table 2:** Follow up data

	Mean	SD	n
Child measurements			
ADHD-RS reduction	− 12.15	6.46	64
ADHD-RS baseline	43.11	12.53	53
ADHD-RS 3 months	37.36	12.24	61
ADHD-RS 6 months	34.56	13.33	59
ADHD-RS 9 months	31.31	13.45	59
ADHD-RS 12 months	30.25	14.44	57
Parent measurements			
ASRS 1.1	28.05	14.57	59
RSA	146.07	31.12	57
ECR	166.75	40.11	56

The ADHD-RS reduction is a linear prediction from a mixed effects model. This a reflection of the observed average reduction from baseline to the 12 months follow up but allowing for missing data points caused by non-attendance

*ADHD-RS* ADHD-Rating Scale, *ASRS 1.1* Adult ADHD Self-Report Scale, *RSA* Resilience Scale for Adults, *ECR* Experiences in Close Relationships

The ECR, RSA and ASRS scores used are latent variable predictions derived from structural equation models. ECR and RSA items are modeled assuming multivariate normality. ASRS items are considered ordinal items and modeled using a logit link function. All three latent variable predictions are standardized with means at 0 and variances fixed to 1. The latent variable approach has the advantage of removing the residual (unexplained by the latent factor) variance for each item when predicting the latent variable. Measurement error associated with psychometric scales is minimized in this way.

Because of some missing data on covariates, we used a full information maximum likelihood estimator implemented in Stata 14. Means and variances as well as covariance for exogenous variables were estimated and used as the basis for corrected regression estimates. Missing data due to non-attendance at the follow up measurements were assumed to be random, i.e. not associated with the unobserved outcome measurement. This assumption was substantiated by a missing data analysis showing no significant association between baseline ADHD-RS score and missing data (RR = 1.02, p = 0.47) and between the ADHD-RS score preceding (t_−1_) a missing data incident (RR = 1.01, p = 0.66). Mixed effects Poisson models were used for the missing data analysis.

## Results

In the follow up sequence, 92.2% (N = 59) had a maximum of one missed self-report.

Whilst all fathers were invited to participate and all consented to do so, only a minority actually completed the questionnaires (22.4%). Consequently, the paternal response rate was too low to include for further analysis. Complete information from all planned assessments was obtained for 62.5% (N = 40) of the children. Two children were only measured twice from baseline to the 12 month follow up. Table 2 presents the descriptive results, including means, standard deviations and number of observations for ECR, RSA and ASRS as well as ADHD-RS at baseline, follow up and the overall ADHD-RS reduction as predicted in a random effects regression. Correlations are provided in Additional file 1: Table S1. ADHD-RS was on average reduced by 12.15 points as predicted in the random effects regression. The mean ADHD-RS at baseline was 43.1 and reduced to 30.25 one year after initiating treatment.

### Predicting ADHD-RS reductions

The following demographic variables were included in the analysis: gender, age, family history of mental illness and whether the child is living in a one or two parent household. A binary indicator of medical treatment and the number of non-attendances were also included as covariates. Table 3 show regression estimates for the association between maternal ECR, RSA, ASRS and the change in child ADHD-RS over the 12 months of follow up. Adjusted and unadjusted results are shown. The negative association between RSA and the outcome (b = − 1.76; 95% CI − 3.45, − 0.07) suggests that a higher degree of maternal resilience is associated with larger reductions in child ADHD-RS during treatment. The significant association between maternal ASRS and child ADHD-RS (b = 3.48; 95% CI 1.76,5.20) suggests that children of mothers scoring higher on ADHD symptoms achieved a more modest treatment effect than children of mothers scoring lower on ADHD symptoms. There was no significant association between maternal ECR and the change in child ADHD-RS. The coefficients correspond to the predicted absolute change in ADHD-RS from baseline to 12 months follow up when ECR, RSA or ASRS increase by one standard deviation.

**Table 3:** Overall ADHD-RS reduction predicted by ECR, RSA and ASRS

	Crude		Adjusted	
	b	95% CI	b	95% CI
ECR	− 1.68	[− 3.38, 0.02]	− 1.18	[− 2.91, 0.55]
RSA	− 1.98	[− 3.66, − 0.31]*	− 1.76	[− 3.45, − 0.07]*
ASRS	3.44	[1.78, 5.10]***	3.48	[1.76, 5.20]***
Control variables				
Female (ref. male)			0.30	[− 3.06, 3.66]
Age at baseline			− 0.29	[− 1.55, 0.98]
Split family (ref. nuclear)			0.09	[− 2.88,3.07]
In medical treatment (ref. not)			− 5.32	[− 8.79, − 1.84]**
Psych. disp. (no psych. Disp.)			− 1.21	[− 5.71,3.29]
Missed out follow ups			0.30	[− 1.86,2.46]

N = 64. Linear regression using full information maximum likelihood estimator. ECR, RSA and ASRS are standardized factor scores used only in separate models. Outcome is the reduction in ADHD-RS score during 12 months follow up. It is predicted in a mixed effects regression including all information available. Crude and adjusted estimates are controlled for baseline ADHD-RS

*ASRS 1.1* Adult ADHD Self-Report Scale, *RSA* Resilience Scale for Adults, *ECR* Experiences in Close Relationships

* p < 0.05, ** p < 0.01, *** p < 0.001

## Discussion

The current study was undertaken with the broad aim of attempting to shed further light on the role of maternal functioning in the treatment response of children diagnosed with ADHD. Informed by the literature and existing empirical findings we chose to evaluate three domains of potential importance in maternal functioning: self-reported ADHD symptoms, self-reported attachment style and degree of resilience. A sample of sixty-seven mother–child dyads with ADHD-diagnosed children was recruited. The children and families received care as usual, and treatment response was evaluated in terms of ADHD symptom-load measured at 3 monthly intervals over a 1-year period. We anticipated that all three maternal functioning factors would correlate with treatment response, independent of treatment strategy.

The findings suggested a significant association between maternal self-reported ADHD symptoms and treatment outcome, measured by a reduction in children’s reported ADHD symptoms. Thus, mothers scoring high on ADHD symptoms had children who showed a lower reduction in ADHD symptoms at the 12 months’ follow up.

Contrary to expectations, we found no significant correlation between maternal self-reported attachment style on the ECR and child outcome on the ADHD-RS.

Lastly and in line with our prediction, we found a negative association between maternal resilience as measured by the RSA and offspring treatment response on the ADHD-RS. This suggested that a higher degree of resilience in mothers was associated with greater symptom-reduction in their children receiving care as usual.

The correlation between maternal ADHD symptomatology and treatment outcome of children with ADHD may be increased level of conflict in the parent–child relationship and exacerbated negative parenting [52]. Harvey and colleagues found that parental ADHD symptomatology was associated with a number of factors in parenting practices and quality of parent–child interactions. This is supported by the findings of a number of studies, suggesting that maternal psychiatric history is significantly associated with child symptom severity and that there is a need for parental screening and treatment programs developed specifically for families in need [53, 54].

Regarding ADHD and attachment, these have been found to constitute mutual risk factors and show extensive overlap in symptomatology, [55]. However, our results did not support a correlation between maternal attachment style and treatment outcome in offspring ADHD. Notably, the ECR assesses attachment-related thoughts and feelings in adult romantic relationships and belongs to the social psychological tradition, whereas instruments such as the Adult Attachment Interview (AAI) represent the developmental assessment tradition [56]. These alternative traditions in assessing attachment have been found to be only partly overlapping [57]. However, we have assessed the mother–child dyads using the AAI as well, and found that the AAI did not correlate with short-term treatment outcome either [58]. One explanation for this unexpected finding may be that maternal psychopathology is a more important predictor than attachment representations in offspring treatment outcome.

In terms of risk and resilience, numerous studies have documented the relationship between parental risk factors and child development. For example; association between family structure and mental wellbeing of children (rate of readmissions to hospital) were investigated and pointed to the significance of family trauma and family psychiatric history [59]. Further, in a meta-analysis of clinical samples, maternal functioning was found to be more important than factors in the child in shaping the quality of infant-mother attachment relationship [60].

Resilient child development despite adversity and how to promote resilient parenting are topics of increasing interest [61–63]. Surprisingly, however, few studies to date have investigated the potential influence of maternal resilience on treatment outcome in children with psychopathology, and specifically those diagnosed with ADHD. Our findings suggest that maternal resilience may be a significant factor in predicting how the child diagnosed with ADHD responds to treatment. In line with this, a study on parenting practices found the combination of family risk, protection and parenting practices to be highly predictive of child functioning [64]. This underlines the need to consider resources as well as risk factors.

As resilience is generally regarded as a more stable trait than psychiatric symptoms [65] it may prove to be a powerful predictor and a core maternal feature in overall prognosis in children affected by various psychiatric symptoms.

Some studies in ADHD treatment have focused on parenting training and social skills training for children with ADHD. However, the evidence has not been convincing [66–69] which may result from lack of focus on maternal functioning, such as own unmet need for treatment [70]. This omission in the field needs to be addressed by future studies placing greater emphasis on examining the relationship between maternal functioning and child treatment in the prediction of developmental outcomes. This is underlined by the fact that our findings were significant in the domains of maternal ADHD symptoms and resilience regardless of treatment strategy, as not all children received medical treatment. Factors in maternal functioning may potentially provide a basis for more differentiated treatment strategies in the future. This may in turn improve the general prognosis in ADHD.

### Methodological considerations

This study is unique in representing the first attempt at exploring the role of maternal functioning in predicting treatment response of children with ADHD; an area which has been lacking in previous studies [15, 16].

The study is, to our knowledge, one of very few studies to address maternal resources as well as risk factors simultaneously and it clearly raises important questions for future research.

Nevertheless, the current study has several limitations.

Our study had a naturalistic design and applied consecutive recruiting in order to include and follow a sample representative of the population we intended to investigate. This was to keep selection bias to a minimum, and hence, we further kept the research track separated from the clinical treatment during the 1-year follow up. However, since a little more than half declined to participate, this then resulted in selection bias. When asked, the participant’s main reason for declining to participate was due to the stress of the many appointments entailed by the clinical track.

Further, another limitation relates to the duration of the follow up period. As factors in the parent–child relationship are likely to emerge over time, a longer follow up would have been ideal.

For example, the lack of correlation between maternal self-reported attachment style and child outcome on ADHD-RS may relate to the relatively short follow up period. This is supported by findings from other studies suggesting that the influence of medical treatment tends to wear off, making other factors more influential in long-term treatment strategies [71, 72].

Another limitation concerns the sole reliance on self-reporting in the assessment of various aspects of maternal functioning as socially desirable responding has been shown to affect results in previous research [73]. However, it has been shown that adults provide an accurate assessment of their own ADHD symptoms [74].

Also, maternal ADHD symptomatology was assessed very specifically; other types of maternal psychopathology were not assessed and thus taken into consideration. For example, as maternal depression is known to be a risk factor for symptom severity in offspring ADHD [19], it would have been of interest to gain information on how many mothers were clinically depressed. We did, however, obtain general information on history of mental illness, from preformed protocols collecting psychosocial and demographic data, which was controlled for in the analysis. The same is also true of the assessment of the children, as the degree of impairment in the children was not assessed beyond an assessment of their ADHD symptoms.

Disappointingly, we were not able to draw any conclusions in relation to the role of fathers’ functioning in the treatment response of their children diagnosed with ADHD. The response rate of fathers was too low to include in the study. Whilst this is not unique to our study (see for example, [75]), previous findings do suggest a link between child externalizing problems and paternal ADHD symptoms, and this may constitute an area of particular interest for future research [54].

Compared to other reported studies, the rate of comorbidity was very low in our study [76, 77]. However, it would appear that ADHD is a clinical predictor of comorbidities, as ADHD seems to increase the risk of developing comorbidity during childhood and adolescence such as conduct disorder and oppositional defiance disorder, which further increase the risk of antisocial behavior and juvenile delinquency later on ([11, 13, 78, 79].This developmental pathway was also observed in a longitudinal study in which children were included only if they had no comorbidity [12]. Yet, in this study by Klein and colleagues, 84 of 135 participants developed probable or definite conduct disorder during adolescence and a further 25% of these developed antisocial personality disorder in adulthood. The children in our study were included after a first time referral and were in the age range 7–12. Hence, the frequency of comorbid disorders may rise in the years to come.

## Conclusion

Taken together, our findings suggest that risk factors as well as protective factors in maternal functioning have an impact on treatment outcome in children with ADHD. This underlines the potential value of a broader assessment of maternal functioning, including screening of mothers for ADHD symptoms. This would permit the identification of parents with unmet needs for treatment and support, which might in turn lead to a better prognosis for their children. On the other hand, a greater focus on protective factors such as maternal resilience may be no less important in differentiating the subgroups of families who are at less of an immediate risk and who may therefore require less support and intervention.

## Additional file

**Additional file 1: Table S1.** Correlation matrix.

## Abbreviations

ADHD: attention deficit hyperactivity disorder
AAI: Adult Attachment Interview
ADHD-RS: ADHD-Rating Scale
ASRS-1.1: Adult ADHD Self-Report Scale
ECR: Experiences in Close Relationships Questionnaire
ECR-R: The Experiences in Close Relationships Scale—Revised
RSA: Resilience Scale for Adults
CT: combined type
IT: inattentive type
ODD: oppositional defiance disorder
