# Supplementary material for: Childhood ADHD and treatment outcome: the role of maternal functioning
Source: Child Adolesc Psychiatry Ment Health. 2018 Jun 14;12:31. doi: 10.1186/s13034-018-0234-3 (PMC6003115; doi:10.1186/s13034-018-0234-3)
Supplement: Supplementary file 1 — Additional file 1: Table S1. Correlation matrix. [file 13034_2018_234_MOESM1_ESM.docx]

| **Table S1:**  **Correlation matrix** | ​ | ​ | ​ | ​ | ​ |
| --- | --- | --- | --- | --- | --- |
| **​** | ADHD-RS  baseline | ADHD-RS  slope | ASRS | Resilience | ECR |
| **ADHD-RS baseline** | 1.00 | ​ | ​ | ​ | ​ |
| **ADHD-RS slope** | -0.10 | 1.00 | ​ | ​ | ​ |
| **ASRS** | 0.48*** | 0.39** | 1.00 | ​ | ​ |
| **Resilience** | -0.22 | -0.25* | -0.51*** | 1.00 | ​ |
| **ECR** | -0.22 | -0.21 | -0.36** | 0.43*** | 1.00 |

* p<0.05, ** p<0.01, *** p<0.001
